# Hazard assessment of *Staphylococcus* with positive coagulase in meat produced and distributed in the Northern regions of Cameroon

**DOI:** 10.14202/vetworld.2019.466-471

**Published:** 2019-03-30

**Authors:** Raoul Bakari Afnabi, Jean Jacques Nenba Sambo, Moctar Mohamed Mouliom Mouiche, Rodrigue Simonet Poueme Namegni

**Affiliations:** 1 Department of Microbiology and Infectious Diseases, Ngaoundere University, School of Veterinary Medicine and Sciences, P.O. Box: 454, Ngaoundere, Cameroon; 2 Division of Microbiology, Institute of Agricultural Research for Development, P.O. Box: 65, Wakwa, Cameroon; 3 Department of Pharmacy-Pharmacology and Toxicology, Ngaoundere University, School of Veterinary Medicine and Sciences, P.O. Box: 454, Ngaoundere Cameroon; 4 Department of Animal Pathology, National Veterinary Laboratory (LANAVET), P.O. Box: 503, Garoua, Cameroon

**Keywords:** contamination, meat, Northern regions of Cameroon, resistance to antibiotics, *Staphylococcus* with positive coagulase

## Abstract

**Aim:**

*Staphylococcus* with positive coagulase (SPC) is a major problem for beef consumers in the northern part of Cameroon. For this purpose, the SPC concentrations in beef produced and supplied in the northern regions were determined, as well as the resistance profile of these bacteria to antibiotics.

**Materials and Methods:**

A total of 125 samples were obtained by the wet and dry swabbing method in traditional slaughterhouses and butcheries to evaluate the SPC concentration in meat, and then, 102 SPC isolations were collected to determine the antibiotic resistance profile.

**Results:**

The distribution of concentration of the SPC indicated no significant differences of bacterial evidence in almost all the slaughterhouses except the one in Manwi (with 2.66 log CFU/cm²) and the density in SPC is higher than that one in Guider (1.99 log CFU/cm²). The assessment of density in SPC among the selected slaughterhouse highlighted a superiority of the SPC concentrations in the Ngaoundere butcheries (3.83 log CFU/cm²) in comparison with those of other towns. At the level of the slaughterhouses, a higher proportion of resistance to Penicillin G was recorded than Gentamicin. Some butcheries recorded that all SPC reacted to Kanamycin, whereas they were more resistant to Penicillin G.

**Conclusion:**

These results reveal that the SPC found in meat poses a threat to meat consumers in the northern part of Cameroon.

## Introduction

The food security of beef is a major concern in Sub-Saharan Africa, including Cameroon [1], wherein beef is the main source of animal proteins for human consumption. Beef is also considered as a high-risk food product because it harbors some pathogenic microorganisms [2]. In general, human microflora originates from various food intakes [3] with some bacteria which alone represent the two-thirds of total microflora [4]. Among other infectious microbes, *Staphylococcus* are known as the main cause of toxi-infections among humans of all age groups [4]. Several *Staphylococcus* intoxication cases were reported in the world. A clear example is a case study carried out in La Loire between 1996 and 2013, wherein members of the 767 households were infected, 211 cases are caused by *Staphylococcus* [4], and the other study carried out between 2011 and 2012 where the *Staphylococcus* play a great part with the bacteria responsible for food intoxication in Morocco with 31% [5]. *Staphylococcus* resistance to antibiotics is another emerging problem. Many cases of *Staphylococcus* resistance to antibiotic are reported throughout the world, namely, methicillin resistance found in 264 of examined meat samples, for example, 11.9% of the 2217 samples chosen most of which came from veal meat (15.2%) and beef (10.6%) [6].

In other studies carried out in the USA over 136 samples of meat and poultry, 96% of isolated *Staphylococcus aureus* were resistant to at least one type of antibiotic [7]. The dangers related to the consumption of meat contaminated with *Staphylococcus* justify the studies carried out on these bacteria. Studies were carried out on this topic in 2012 in the Cotonou-Porto-Novo slaughterhouses [8], the ones carried out the same year in the butcher’s block of Kumbungu and Tolon towns [9] and the last results in the Ouagadougou slaughterhouses [10].

However, no study has explored the domain of beef contamination germs in Cameroon. The aim of this study was to assess the dangers associated with the *Staphylococcus* with positive coagulase (SPC) on raw beef produced and supplied in the northern regions of Cameroon. It is specifically based on the evaluation of the SPC densities of the meat produced and distributed in the northern part of Cameroon and the specification of the resistance profile of the isolated SPC to antibiotics.

## Materials and Methods

### Ethical approval

Samples were collected under the supervision of the Veterinary Services and microbiological analysis was carried out at the National Veterinary Laboratory.

### Sampling

This study was carried out in three phases from March to June 2014. The samples were collected at the slaughterhouses and butcheries in the study areas Manwi, Guider, Pitoa, Garoua, and Maroua towns. Altogether, 125 samples were collected where 71 of them were from the slaughterhouses and 54 from the butcheries and their respective suppliers.

The samples were collected using previously published wet and dry swabbing method [11] on the surface of the carcasses (slaughterhouses) and the quarters (butcheries). A total of 982 swabs were collected in this study divided between 568 swabs from the slaughterhouses and 414 swabs from the butcheries. The surfaces corresponded to neck, thorax, flank, and rumsteck [11,12]. The sampling phases were just collected after a veterinary inspection in some slaughterhouses while in the butcheries, the time slot was 9:30 AM-10:30 AM right after the carving operations. The swabs were kept refrigerated (0-4°C) in an ice box containing some ice packs. The box was equipped with a thermometer to monitor the samples shipping temperature. Afterward, the sampling box was transported to the National Veterinary Laboratory in Boklé Garoua.

### Microbiological analysis

All the samples were tested 24 h after the sampling phases proper. The discovery and the counting of the SPC were conducted according to the French norms (EN V08 057-1) [13]. To achieve the antibiogram, a total of 102 strains of SPC were used in this study. 56 strains came from slaughterhouses and 46 others came from butcheries. The methods used for the realization of the classical antibiogram and to test the sensibility of the SPC to methicillin were described by the dissemination method over the Mueller-Hinton oversalted agar [14]. In addition, antibiotics such as Penicillin G, Gentamicin, Kanamycin, Oxytetracycline, Trimethoprim-sulfonamide, and Nitrofuran were used for antibiogram experimentation.

### Statistical analysis

The XL-Stat software was used for the statistical analysis. The Mann–Whitney U-test helped to compare the frequency of contaminations caused by the SPC between the slaughterhouses and the butcheries. Moreover, the relations existing between the antibiotic resistance profiles and the structures in charge of the sampling origins (slaughterhouses and butcheries) were examined by the χ^2^ test (Σ²).

## Results

### Meat microbiological contamination

#### Slaughterhouses

The densities of *Staphylococcus*, respectively, varied from 1.97±0.8 log (CFU/cm²) to 2.6±0.66 log (CFU/cm²) (Table-1). The comparison in the distribution of the loads between the various slaughterhouses disclosed a no significant difference of these densities in almost all the totality of those slaughterhouses; except the Manwi and Guider slaughterhouses which showed a significant difference (p=0.03 <α) in the distribution of their microbial loads (Table-2).

**Table-1:** Mean concentration of *Staphylococcu*s with positive coagulase in some slaughterhouses.

Traditional slaughterhouse (log (CFU/cm²)	MSK n=15	MSG n=15	MSM n=11	MSP n=15	MSGd n=15
Carcass_1_	4.07±1.13	2.56±0.12	3.72±0.79	1.53±0.31	1.62±0.26
Carcass_2_	2.1±0.17	2.27±0.07	2.28±0.13	2.47±0.97	2.87±0.61
Carcass_3_	1.49±0.61	1.84±0.39	1.68±0.43	2.02±1.36	2.57±0.44
Carcass_4_	3.57±0.81	2.37±0.04	3.19±0.45	0.8±2.33	2.04±0.1
Carcass_5_	0.97±0.95	3.42±0.7	2.47±0.01	2.97±0.99	1.52±0.26
Carcass_6_	1.1±0.96	2.68±0.24	2.64±0.13	2.39±1.49	1.62±0.21
Carcass_7_	3.86±0.88	1.84±0.33	2.38±0.03	2.01±1.9	2.66±0.5
Carcass_8_	2.1±0.25	3.49±0.8	3.15±0.51	2.01±2.12	0.97±0.63
Carcass_9_	1.1±0.99	1.57±0.44	1.71±0.33	1.82±2.52	3.1±0.78
Carcass_10_	2.75±0.01	1.81±0.35	3.16±0.52	1.44±3.14	2.51±0.49
Carcass_11_	4.36±1.15	3.16±0.53	1.69±0.00	2.12±3.19	1.77±0.07
Carcass_12_	1.94±0.27	2.41±0.14		0.22±5.17	1.01±0.45
Carcass_13_	2.82±0.26	2.8±0.46		1.97±5.22	2.7±0.6
Carcass_14_	2.53±0.19	1.97±0.11		3.52±5.87	1.22±0.15
Carcass_15_	2±0.00	1.67±0.00		2.32±9.65	1.65±0.00
Means±SD	2.45±1.11	2.39±0.63	2.6±0.66	1.97±0.8	1.99±0.7

MSK=Municipal slaughterhouse of K-djidéo, MSG=Municipal slaughterhouse of Garoua, MSM=Municipal slaughterhouse of Manwi, MSP=Municipal slaughterhouse of Pitoa, MSGd=Municipal slaughterhouse of Guider, SD=Standard deviation

**Table-2:** Comparison of loads of *Staphylococcu*s with positive coagulase among the slaughterhouses.

Slaughterhouses	K-djidéo	Garoua	Pitoa-Garoua	Guider
Garoua	0.91			
Pitoa-Garoua	0.30	0.22		
Guider	0.29	0.12	0.98	
Manwi	0.65	0.38	0.05	0.03*

(*p<0.05; ANOVA de Mann–Whitney)

#### Butcheries

*Staphylococcus* concentration varied between 2.32±1.5 log (CFU/cm²) for butcheries receiving meat from the Garoua slaughterhouse and 3.82±1.01 log (CFU/cm²) for those which are supplied by those in Ngaoundere (Table-3). The difference in the distribution of the *Staphylococcus* among these butcheries revealed a significant difference of the SPC densities to Ngaoundere town, assessments of the concentration of *Staphylococcus* were rather not significant (Table-4).

**Table-3:** Mean of *Staphylococcus* with positive coagulase in the butcheries.

Butcheries log (CFU/cm²)	B[MSK] n=12	B[MSG] n=9	B[MSM] n=12	B[MSP] n=9	B[MSGd] n=12
Carcass_1_	4.69±1.32	1.4±0.65	3.99±0.28	2.61±0.04	2.35±0.36
Carcass_2_	3.18±0.37	0.57±1.32	4.03±0.34	2.52±0.1	2.67±0.17
Carcass_3_	2.71±0.08	4.29±1.13	5.26±1.24	2.42±0.18	3.04±0.08
Carcass_4_	2.65±0.04	2.49±0.04	3.97±0.47	1.48±0.88	3.9±0.69
Carcass_5_	3.24±0.47	2.34±0.06	2.58±0.45	2.15±0.59	3.05±0.18
Carcass_6_	2.05±0.31	4.05±1.13	5.03±1.21	2.48±0.5	3.18±0.3
Carcass_7_	2.55±0.01	0±1.36	1.98±0.75	2.62±0.57	2.79±0.07
Carcass_8_	3.8±0.87	2.11±0.54	3.94±0.49	3.46±0.26	1.64±0.73
Carcass_9_	1.1±0.82	3.64±0.00	2.19±0.62	4.19±0.00	2.07±0.6
Carcass_10_	2.24±0.29		2.99±0.27		4.66±1.03
Carcass_11_	3.24±0.28		3.64±0.06		1.64±0.59
Carcass_12_	2.46±0.00		3.47±0.00		3.31±0.00
Mean±SD	2.82±0.9	2.32±1.5	3.83±1.01	2.65±0.77	2.86±0.88

B[MSK]=Butchery associated with the Municipal slaughterhouse of K-djidéo, B[MSG]=Butchery associated with the Municipal slaughterhouse of Garoua, B[MSM] =Butchery associated with the Municipal slaughterhouse of Manwi, B[MSP]=Butchery associated with the Municipal slaughterhouse of Pitoa, B[MSGd]=Butchery associated with the Municipal slaughterhouse of Guider, SD=Standard deviation

**Table-4:** The SPC load comparisons among the butcheries.

Butcheries	Maroua	Garoua	Pitoa-Garoua	Guider
Garoua	0.39			
Pitoa-Garoua	0.52	0.45		
Guider	0.93	0.39	0.48	
Ngaoundere	0.01*	0.04*	0.01*	0.02*

(*p<0.05, ANOVA de Mann–Whitney), SPC=*Staphylococcus* with positive coagulase

### Distribution of Staphylococcus contamination

Every bar chart represents the average of the standard deviation; p=0.05.

### Antibiotic resistance to the SPC

#### Slaughterhouses

The evaluation of the resistance to the identified SPC in slaughterhouses showed a variation in resistant profiles. The resistance to Penicillin G was the highest proportions while the resistance to Gentamicin and Furan was the lowest proportions (Table-5). The χ^2^ test analysis done revealed that the antibiotic resistance profiles varied according to slaughterhouses (p=0.025; α=0.05; p<α).

**Table-5:** Resistance to *Staphylococcus* with positive coagulase.

Slaughterhouses	K-Djidéo n (%)	Guider n (%)	Pitoa n (%)	Garoua n (%)	Manwi n (%)
Penicillin G	10 (71.42)	12 (66.67)	8 (80)	3 (42.85)	2 (28.57)
Gentamicin	0	0	0	0	0
Oxytetracycline	2 (14.28)	3 (16.67)	1 (10)	0	4 (57.14)
Trimethoprim+Sulfonamide	1 (7.14)	1 (5.56)	0	0	0
Nitrofuran	1 (7.14)	0	0	0	1 (14.28)
Kanamycin	0	2 (11.12)	0	0	0
Methicillin	4 (28.57)	1 (5.56)	1 (10)	0	1 (14.28)

#### Butcheries

Irrespective of the butchery, all SPCs were sensitive to Kanamycin, but most were resistant to Penicillin G (Table-6). The χ^2^ test revealed that the antibiotic resistance profiles varied according to the butcheries (p=0.003; α=0.05; p<α).

**Table-6:** Resistance to *Staphylococcus* with positive coagulase in the butcheries.

Butcheries	K-Djidéo n (%)	Guider n (%)	Pitoa n (%)	Garoua n (%)	Manwi n (%)
Penicillin G	7 (63.64)	10 (55.56)	3 (50)	3 (60)	4 (66.67)
Gentamicin	1 (9.1)	0	0	0	0
Oxytetracycline	2 (18.18)	8 (44.44)	6 (100)	2 (40)	2 (33.33)
Trimethoprim+Sulfonamide	1 (9.1)	1 (5.56)	0	0	0
Nitrofuran	0	0	2 (33.33)	0	0
Kanamycin	0	0	0	0	0
Methicillin	2 (18.18)	2 (11.11)	2 (33.33)	1 (20)	2 (33.33)

## Discussion

Results showed that the SPC loads were almost closer in all the slaughterhouses studied with the exception of the Manwi municipal slaughterhouse where the load of SPC was much higher than those of Guider and K-djidéo. However, the environmental contamination could explain this point for the Manwi warehouse was not surrounded with used fence and its surface was damaged, badly, or not cleaned [15]. In addition, the water used to clean the carcasses and the equipment stemmed from a surrounding watercourse and of doubtful sanitary origin. This type of meat contamination by the environment was highlighted in the Cotonou-Porto-Novo slaughterhouse in Benin [16]; the SPC load was lower than the medium threshold value, however, showed a similarity with those reported at the traditional slaughterhouses in Tiaret (Algeria) (2.15 log CFU/cm², 2.93 log CFU/cm²) [17]. This could be justified by the least respect of the hygiene rules because the presence of *Staphylococcus* on the carcasses would be either through physical contact or indirect contact (water air, equipment, staff, etc.) [18,19]. Consequently, fresh meat obtained from SPC would probably be the cause of *Staphylococcus* toxi-infection among the consumers.

The SPC concentrations were higher in the Ngaoundere butcheries than in the other towns, but no significant difference was observed in the other butcheries of the other towns. In the different butcheries in Ngaoundere, the higher concentration of SPC would probably be due to the higher number of people manipulating meat at the level of the trading posts for the human being harbors different types of bacterial flora that could be a source of significant food contaminations [20]. The presence of SPC detected in the various slaughterhouses of the northern regions of Cameroon was of same magnitude like what was obtained in the Kumbungu and Tolon towns in Ghana; hence, justifying the similarity in the unhygienic practices observed in these areas where meat is sold [9,21]. The butchers were no more respecting the basic rules related to the cleaning up of hands before and after manipulating meat (during transport slicing processes), the non-conditioning of meat in packages and the regular disinfecting of the trading posts usage [22,23]. These non-conformities of hygienic rules with regard to the butchery norms lead to the increase in the growth and the proliferation of the SPC responsible for toxi-infection of *Staphylococcus* rate in the consumers.

**Figure-1:**
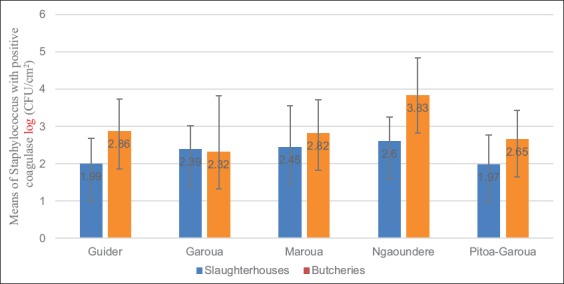
Distribution of the average loads of *Staphylococcus* with positive coagulase.

The no significant difference found in almost all distribution systems compared to production systems (Figure-1). This could be explained by similar practices of manipulation of the raw meats. Moreover, the people who treated raw meats should have similar hygienic practices. Similar results were observed in the region of South Kivu in the Democratic Republic of Congo in 2015 [23].

The relation existing between antibiotic resistance profiles and the slaughterhouses could be due to the source of livestock in each slaughterhouse. Studies carried out by Lozano and Igbinosa revealed that the source of meat contamination by SPC in slaughterhouses would come from microbes on the external hides of slaughtered animals [24,25]. From there, the variety of resistant profiles would probably be the consequence of the germs which were subjected to different conditions of the acquisition of their resistance to antibiotics. Penicillin G and Oxytetracycline were the most representative resistances in all studied slaughterhouses. Similar results were found in Ethiopia [26]. As for the Oxacillin, in Ethiopia, the resistance rate was not superior to that one obtained [26]. This could not explain the important use of that molecule. However, the resistance to antibiotics due to *S. aureus* of meat in some slaughterhouses shows the rise of the danger of those bacteria; especially, the resistance of *S. aureus* to the Oxacillin which is also resistant to all the Penicillin group [27].

As in the slaughterhouses, the strong relationship between the SPC resistance profiles and the butchers would implicate the effect of meat contamination by the distribution chain workers. People involved in the selling of meat could in one way or the others contaminate the meat through their SPC, transmitting at the same time germs that are already resistant in man [28]. From there, the SPC-resistant profiles noticed in the butcheries of the greater North of Cameroon would simply reflect a mixing of *Staphylococcus* having predominantly acquired their resistance in man. On the other hand, the resistance profiles of the SPC samples were higher for the Penicillin G, the Oxytetracycline, and The Oxacillin in the butcheries. Some similar results were also found in the beef and commercialized avians in Quebec [29] and Lumbumbashi [30]. In the slaughterhouses, the resistance to *Staphylococcus* might have originated from animals slaughtered, but in the distribution of meat, the importance of the probable transmissions of resisting germs between people and meat proves that the peak of resistance obtained could be the consequence of a frequent usage of antibiotic molecules for the treatment of patients at some hospitals of Cameroon [31].

## Conclusion

Higher concentrations of SPC in beef in the slaughterhouses and butcheries of the northern regions of Cameroon were detected on the one hand, and on the other hand, high resistance to frequently used veterinary antibiotics such as Penicillin G, Oxytetracycline, and Oxacillin was also detected. The results highlight that the risk incurred by the beef consumers in the absence of strict hygiene rules and preventive methods in traditional production and distribution of that protein sources from animal origin.

## Authors’ Contributions

This study was designed, organized, and supervised by RBA, JJNS and RSPN performed sampling and laboratory analysis. RBA and JJNS analyzed and interpreted the data, while the preparation of the manuscript was done by MMMM, JJNS, and RBA. All authors have read and approved the final manuscript.
